# Evidence for equal size cell divisions during gametogenesis in a marine green alga *Monostroma angicava*

**DOI:** 10.1038/srep13672

**Published:** 2015-09-03

**Authors:** Tatsuya Togashi, Yusuke Horinouchi, Hironobu Sasaki, Jin Yoshimura

**Affiliations:** 1 Marine Biosystems Research Center, Chiba University, Kamogawa 299-5502, Japan; 2 Department of Mathematics and Informatics, Faculty of Science, Chiba University, Chiba, 263-8522, Japan; 3 Graduate School of Science and Technology, and Department of Mathematical and Systems Engineering, Shizuoka University, Hamamatsu 432-8561, Japan; 4 Department of Environmental and Forest Biology, State University of New York College of Environmental Science and Forestry, Syracuse, NY 13210, USA

## Abstract

In cell divisions, relative size of daughter cells should play fundamental roles in gametogenesis and embryogenesis. Differences in gamete size between the two mating types underlie sexual selection. Size of daughter cells is a key factor to regulate cell divisions during cleavage. In cleavage, the form of cell divisions (equal/unequal in size) determines the developmental fate of each blastomere. However, strict validation of the form of cell divisions is rarely demonstrated. We cannot distinguish between equal and unequal cell divisions by analysing only the mean size of daughter cells, because their means can be the same. In contrast, the dispersion of daughter cell size depends on the forms of cell divisions. Based on this, we show that gametogenesis in the marine green alga, *Monostroma angicava*, exhibits equal size cell divisions. The variance and the mean of gamete size (volume) of each mating type measured agree closely with the prediction from synchronized equal size cell divisions. Gamete size actually takes only discrete values here. This is a key theoretical assumption made to explain the diversified evolution of isogamy and anisogamy in marine green algae. Our results suggest that germ cells adopt equal size cell divisions during gametogenesis.

Differences in sperm and egg size are evident in many animals and land plants^1^. However, variable mating systems are also found in green algal taxa: 1) isogamy, where gamete sizes are identical between the two mating types, 2) slight anisogamy, where the sizes of male and female gametes are slightly different, and 3) marked anisogamy, where their sizes are markedly different^2^,^3^. Anisogamy is considered the first step in the establishment of oogamy with sperm and eggs, the extraordinary sexual dimorphism of gametes found in many animals and plants^4^. The difference in gamete size between the two mating types gives rise to morphological and behavioural sexual differences^5^. Both the search ability of male and female gametes^6^ and the resources allocated for zygote development^7^,^8^,^9^ are dependent upon gamete size. Thus, gamete size is a crucial factor for early embryogenesis.

In early embryos of many organisms, zygotes (single cell) generally divide rapidly with almost no growth, the cleavage stage. Consequently, the size of a cell cluster is equal to that of the original zygote^10^. This is one of the major differences from many other forms of cell divisions, in which both the number of cells and the volume of each cell usually increase. Cleavage is regulated by the nucleocytoplasmic ratio (about 1/6)^11^,^12^ and ends at the beginning of zygotic transcription^13^.

In spiral cleavage, one of the four major types of the holoblastic (complete) cleavage, it has been suggested that equal and unequal cleavages are governed by two different mechanisms that lead to the establishment of the D (dorsal) quadrant which serves as a dorsal organizer^14^,^15^. In equal cleavage, observed in many members of the spiralian phyla (e.g. Polyplacophora, Echiuroidea, Anopla and other classes), the first two cytoplasmic divisions produce four macromeres that are indistinguishable from each other. One of these four macromeres becomes the D quadrant^16^. The D quadrant is determined by the specific position in relation to the animal-vegetal inductive interactions that take place between the fifth and sixth cleavage divisions^14^. In contrast, in unequal cleavage observed in Aplacophora, Scaphopoda, Oligochaeta and other classes, one larger cell and the other three smaller cells are produced in the first two cell divisions. These unequal divisions segregate key vegetal factors^17^, and the bigger cell is specified as the D quadrant^16^,^18^. Thus the form of cell divisions and the resulting cell size are key factors in early embryogenesis as well as gametogenesis.

However, it is difficult to evaluate whether cells are divided equally or unequally in many cases. If cell divisions occur asynchronously, cell sizes are affected by the growth of each cell as well as the form of cell divisions. In this case, validation of the form of cell divisions is difficult. Synchronous cell divisions allow a much easier assessment of the forms of cell divisions. However, we should note that synchronous cell divisions do not always mean equal size cell divisions. Even in synchronous cell divisions, we have to measure the volumes of all the daughter cells individually, right after cell division. Also, such a system might produce gametes with discrete size values, making it easy to find locally stable solutions of gamete size in theoretical evolutionally ecology, since we can examine a limited number of evolutionary trajectories^2^.

We took advantage of the synchronous cell divisions during gametogenesis seen in an Ulvophyceae marine green alga, *Monostroma angicava* Kjellmann^19^. This species has a heteromorphic haplodiplontic life cycle^20^, where haploid gametophytes are distinctively different from diploid sporophytes. Multicellular haploid gametophytes are dioecious and monostromatic (i.e. one-cell layered) saccate plants. Each gametophyte vegetative cell is mononucleated and directly becomes a single gametangium in which all resources are used to produce gametes at a time (holocarpic). Gametes are produced through mitotic cell divisions in each gametangium. Male gametes are often slightly smaller than females. Thus, *M. angicava* is considered a slightly anisogamous species. Also in some species of the genus *Ulva* with an isomorphic haplodiplontic life cycle with two-cell layered plants, slightly anisogametes are produced^21^. Their gametogenesis and gamete release are controlled by the sporulation inhibitor and the swarming inhibitor, respectively, that are excreted between the layers of cells^22^. Cell divisions during gametogenesis appear to be synchronized. The ultrastructure and the biochemical properties regulating gamete release have been revealed^23^. In contrast, in *Monostroma angicava*, it has been suggested that gamete release is controlled by an inducer that is excreted from matured gametangia under light^24^.

In this study, we examine whether cells are divided equally or unequally in size during gametogenesis of *M. angicav*a. We compare the distribution of gamete size directly measured with those predicted assuming various ratios of cytoplasmic divisions in each mating type.

## Results

We can predict the size of gametes of each mating type based on size data of gametangia and the number of gametes formed in each gametangium, assuming various forms of cell divisions. By comparing these predictions with the actual data on gamete sizes, we can determine the forms of cell divisions quantitatively, i.e. the ratios of cell division from equal to highly unequal in size (see Methods for more details).

**Figure 1:**
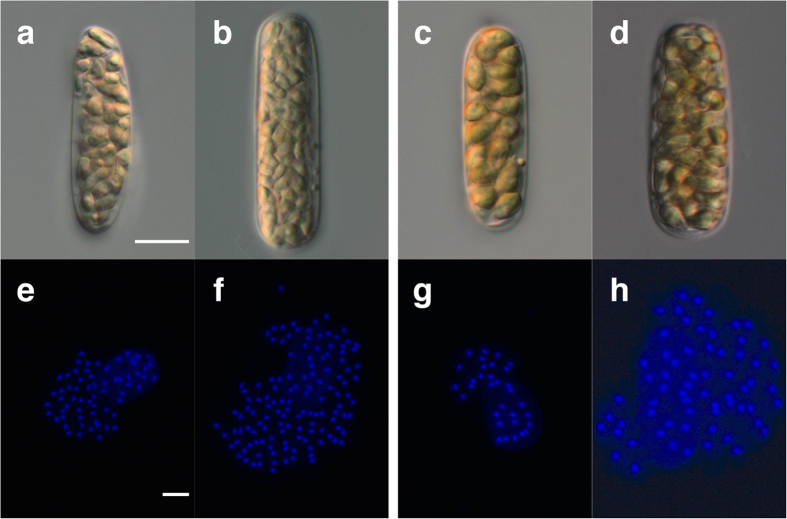
Mature gametangia and DAPI-stained gametic nuclei in each gametangium. (**a**) A male gametangium with 64 gametes. (**b**) A male gametangium with 128 gametes. (**c**) A female gametangium with 32 gametes. (**d**) A female gametangium with 64 gametes. (**e**) 64 gametic nuclei in a single male gametangium. (**f**) 128 gametic nuclei in a single male gametangium. (**g**) 32 gametic nuclei in a single female gametangium. (**h**) 64 gametic nuclei in a single female gametangium. Scale bars = 10 μm.

**Figure 2:**
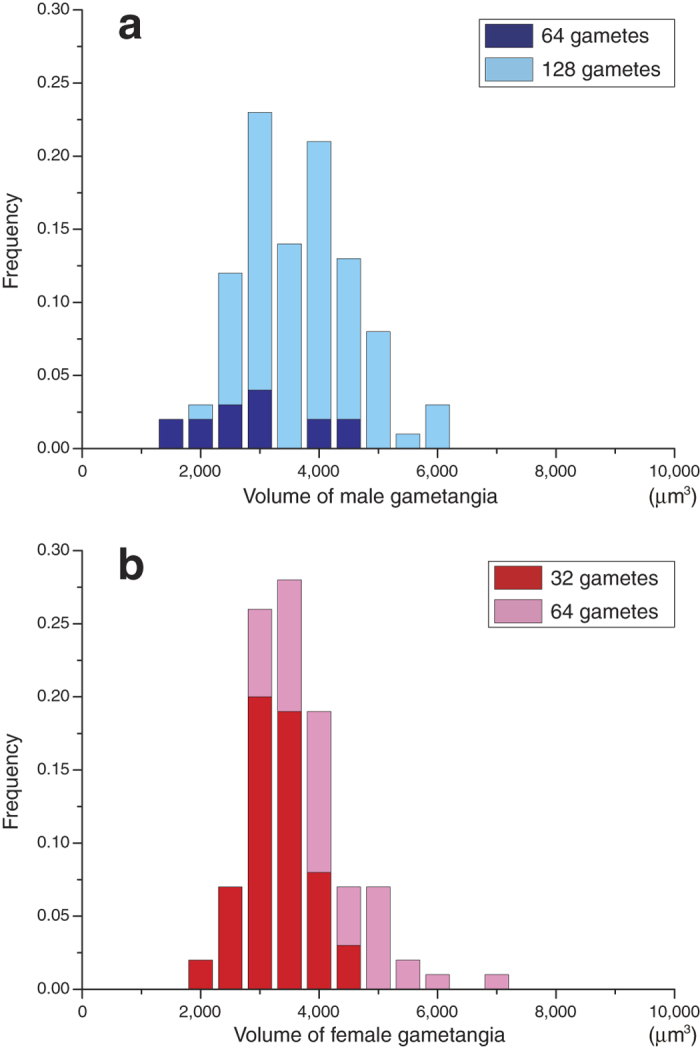
Volume distributions of gametangia for each mating type with the number of gametes formed in individual gametangia. The volume of each gametangium and the number of gametes formed within were examined (*n* = 100 in each mating type). The volume of a gametangium was measured by analysing its vertical cross section on the computer program. (**a**) Male. (**b**) Female. See Methods for more details.

**Figure 3:**
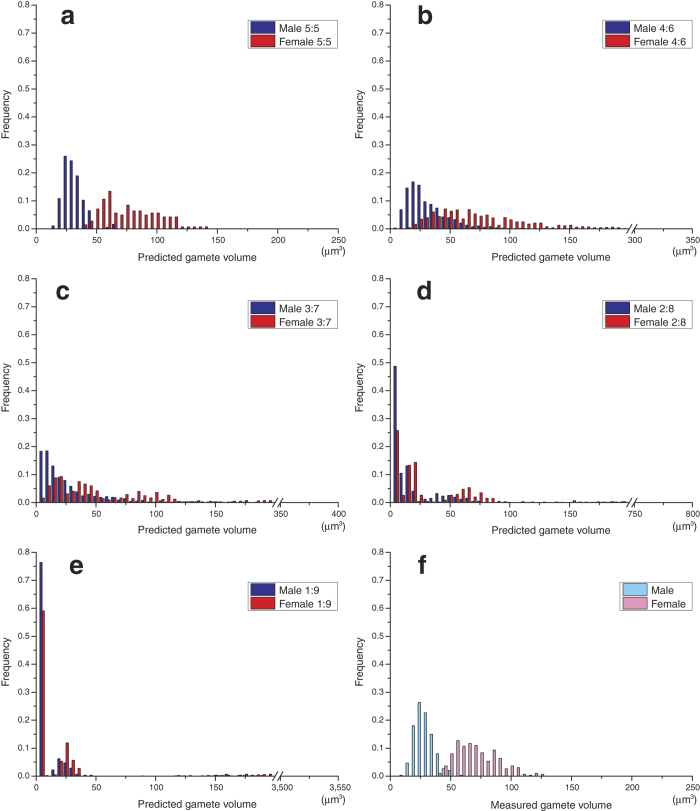
Predicted and measured volume distributions of gametes of each mating type. Gamete volumes predicted based on the number of cell divisions during gametogenesis and the volume of gametangia (Fig. 2) with different ratios of cell divisions (i.e. 5:5 ratio [equal], 4:6, 3:7, 2:8, and 1:9 [unequal]) (male: *n* = 11840 gametes; female: *n* = 4512 gametes), (**a–e**). (**a**) Equal size cell divisions (5:5). (**b**) Unequal size cell divisions (4:6). (**c**) Unequal size cell divisions (3:7). (**d**) Unequal size cell divisions (2:8). (**e**) Unequal size cell divisions (1:9). (**f**) Directly measured gamete volume. *n* = 300 in each mating type.

Separated gametangia are cylindrical in shape (Fig. 1a–d). The distribution of gametangium volumes does not significantly depart from normality in both mating types (male *p* = 0.87, female *p* = 0.26, Chi-square goodness-of-fit test). There is no significant difference between the two mating types in the mean volume of gametangia (*p* = 0.96, Welch’s *t*-test). Gametangia that include different numbers of gametes can be distinguished. Each gametic nucleus in individual gametangia is clearly observed in both mating types (Fig. 1e–h). All nuclei are dispersed, allowing an accurate count. Male gametangia have either 64 (=2^6^; 6 divisions) or 128 (=2^7^) nuclei (Fig. 1e,f, respectively). Female gametangia have either 32 (=2^5^) or 64 (=2^6^) nuclei (Fig. 1g,h, respectively). The volume-distribution histograms of gametangia with the number of gametes formed in individual gametangia show that a larger gametangium tends to contain more gametes in both mating types (Fig. 2). The distributions of volumes of gametangia with different numbers of gametes do not significantly depart from normality in both mating types (male with 64 gametes *p* = 0.14, male with 128 gametes *p* = 0.29, female with 32 gametes *p* = 0.95, female with 64 gametes *p* = 0.46, Chi-square goodness-of-fit test). Gametangia with more gametes are significantly larger than those with fewer gametes in both mating types (male *p* = 0.0032, female *p* = 2.4 × 10^−7^, Welch’s *t*-test). Smaller gametangia of the same size often have different numbers of gametes. Assuming various ratios of cell divisions, we estimate the volume-distribution histograms of gametes in each mating type based on the data (Fig. 2) on the volume of gametangia and the number of cell divisions (Fig. 3a–e). All the predicted volume distributions of gametes depart significantly from normality in both mating types (5:5, 4:6, 3:7, 2:8 and 1:9 ratios, male, *p* ≅ 0, *p* ≅ 0, *p* ≅ 0, *p* ≅ 0, *p* ≅ 0 and female, *p* = 3.4 × 10^−184^, *p* = 3.7 × 10^−232^, *p* ≅ 0, *p* ≅ 0, *p* ≅ 0 respectively, Chi-square goodness-of-fit test). The predicted mean volume of female gametes is significantly larger than that of males (*p* ≅ 0, Mann-Whitney *U* test). The means of predicted gamete volumes within a mating type are the same among all different forms of cell divisions, since the volume of a single gametangium and the number of gametes produced are the same. However, the distributions of predicted gamete volumes differ, depending on the volume ratios of cell divisions.

**Figure 4:**
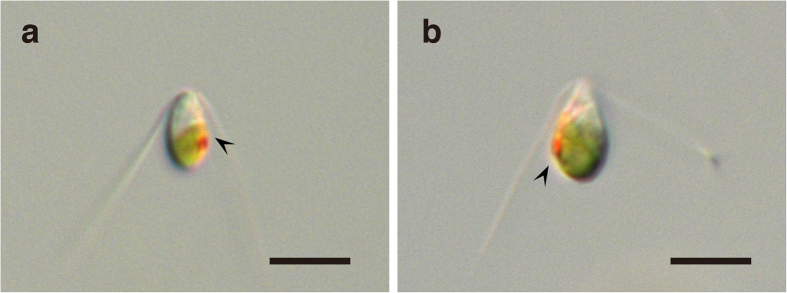
Gametes of *Monostroma angicava*. (**a**) A biflagellate male gamete. (**b**) A biflagellate female gamete. Arrowheads indicate an eye-spot. Scale bars = 5 μm.

Released gametes are similarly pear-shaped in both mating types (Fig. 4). Each gamete has two flagella and an eye-spot. The observed (measured) volume distributions of these released gametes depart significantly from normality in both mating types (male *p* = 0.013, female *p* = 2.8 × 10^−5^, Chi-square goodness-of-fit test). The distributions of the two mating types overlap slightly (Fig. 3f). The mean volume of female gametes is slightly but significantly larger than that of males (*p* ≅ 0, Mann-Whitney *U* test).

**Figure 5:**
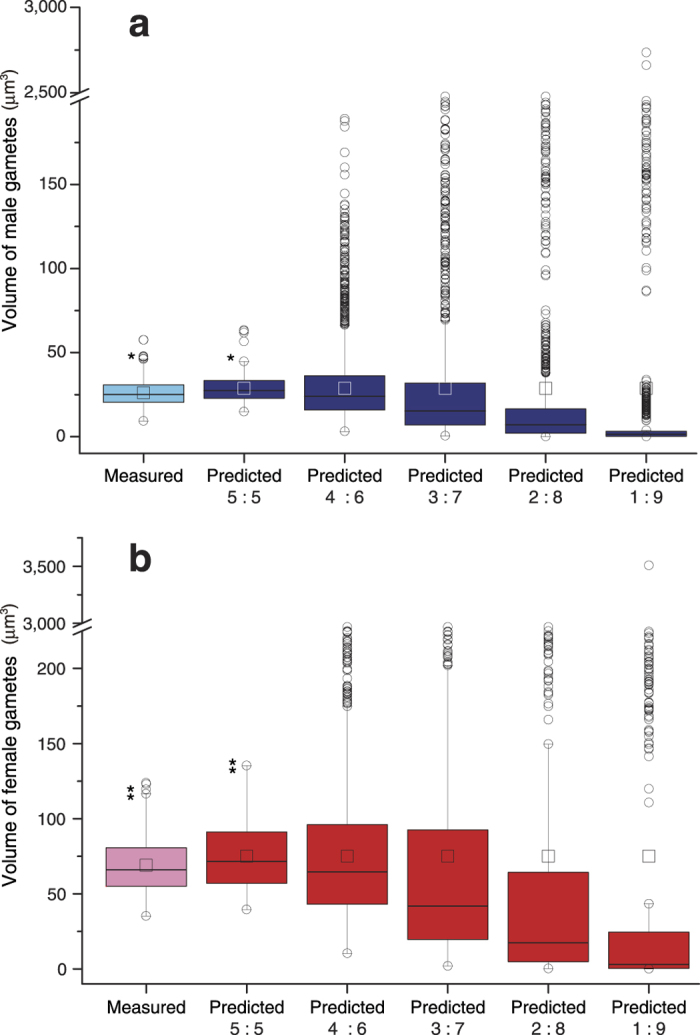
Comparison of variances between measured and predicted gamete volumes with various ratios of cell divisions. Directly measured gametes: *n* = 300 in each mating type. Predicted gamete volumes were calculated under the assumption of equal size cell divisions (5:5 ratio) and unequal (4:6, 3:7, 2:8 and 1:9 ratios) (male: *n* = 11840 gametes; female: *n* = 4512 gametes). Boxes represent the interquartile range (IQR) between first and third quartiles and the line inside represents the median. Whiskers define the lowest and highest values within 1.5 × IQR from the first and third quartiles, respectively. Squares indicate means. Circles represent outliers beyond the whiskers. (**a**) Male. (**b**) Female. We randomly chose 100 gametes in each comparison. *(for male), 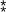
(for female): not significantly different for variance (male *p* = 0.82, female *p* = 0.36, Moses test for equal variability).

We take both of the positions (means) and shapes (variances) into account to compare the predicted and observed distributions. In both mating types, the mean volume of released gametes (direct measurement) is not significantly different from that predicted based on the numbers of cell divisions and the volume of gametangia in cases where cells are equally divided in size (male *p* = 0.88, female *p* = 0.59, Mann-Whitney *U* test). The measured histogram of released gametes (Fig. 3f) is extremely similar to the prediction assuming equal size cell divisions (5:5 ratio; Fig. 3a), but distinctively different from all other predictions that assume unequal size cell divisions (4:6, 3:7, 2:8 and 1:9 ratios; Fig. 3b–e, respectively). To evaluate the similarity between the predicted and observed distributions, we compare the variances of these histograms (Fig. 5). The variance of released gametes is not significantly different from that predicted, assuming equal size cell divisions (5:5) in both mating types (male *p* = 0.82, female *p* = 0.36, Moses test for equal variability see ref. 25 for more details). However, it is significantly smaller than those predicted assuming unequal size cell divisions (4:6, 3:7, 2:8 and 1:9 ratios, male, *p* = 0.00041, *p* = 9.0 × 10^−19^, *p* = 1.9 × 10^−36^, *p* = 4.8 × 10^−40^, and female, *p* = 1.8 × 10^−8^, *p* = 9.0 × 10^−19^, *p* = 2.2 × 10^−22^, *p* = 2.0 × 10^−54^, respectively, Moses test for equal variability).

## Discussion

To examine the form of cell divisions (equal/unequal in size), we are required to analyse the entire distribution of cell size rather than the mean. The numbers of gametic nuclei are always integral positive powers of 2 (Figs 1 and 2). Therefore, the number of gametes in a single gametangium is 2^*n*^, where *n* is the number of cell divisions. We confirm that cell divisions during gametogenesis are strictly synchronized, although the number of cell divisions might be affected by some ecological factors^19^. Thus, the means of cell size may not differ among the different forms of cell divisions (Fig. 3a–e).

The range of cell size arises from variations in the number (times) of cell divisions and the volume of gametangia (Fig. 2). This is why the distribution of gamete size actually departs from normality in both mating types (Fig. 3f). This result is quite different from an important assumption in many previous theoretical models for the evolution of gamete size, in which gamete size of one mating type is treated as a single value (for example, ref. 2).

We should note that all the gametes in *M. angicava* are not atypical but typical with the ability of fertilization (formation of zygotes). The gametes of different mating types fuse irrespective of their sizes^26^. Therefore, gametes of various sizes within one mating type are obviously different from dimorphic gametes known as typical and atypical spermatozoa in various species of animals (e.g. molluscs, insects, echinoderms) where atypical gametes often neither fuse nor develop normally^27^. In this species, there are only two mating types^20^. Our results show that even gametes of the same size would fuse sexually. This means that the terms, isogamy and anisogamy, cannot be strictly applied at the species level. Further, male is defined as the sex that produces smaller gametes and *vice versa*^28^. If we determine the sexes based on the average gamete size, smaller female gametes might fuse with larger male gametes.

The comparison of gametes’ volume distributions indicates that male and female gametes are produced by ‘equally’ dividing the amount of gametic resources in each gametangium during gametogenesis with no significant growth. Our analysis suggests that gamete size values should be discrete in this alga, and gamete size distributions should be taken into account if we develop a theoretical evolutionary model of gamete size based on empirical data. Gametic cells formed in the same gametangium have the same amount of resources in volume as well as the same genes, except for very rare mutation. Particularly, if all gametes in a gametangium have the same genotype, equal cell divisions in size during gametogenesis might be adaptive, because there should be no conflict among gametes over how much cytoplasm they get. These properties in cell divisions during gametogenesis might be observed also in closely related species (e.g. species of the genus *Ulva*) and are quite similar to those supposed in equal size cell divisions of cleavage in early embryogenesis. The current results may imply that the cell divisions in a germ line are in principle equal size cell divisions as long as both daughter cells stay in germ lines. The equal size cell divisions in cleavage can be attributed to the latent expression of this property in the germ line.

## Methods

### Collection of matured gametophytes

Maturation of gametophytes occurs synchronously near the time of spring tides^24^. We collected one pair of mature male and female gametophytes just before synchronized releasing of gametes at a low tide on the Pacific Ocean coast in Muroran, Hokkaido, Japan (42°19′N, 140°59′E). Our sampling site was very close to Muroran Marine Station of Field Science Center for Northern Biosphere, Hokkaido University where we immediately carried out the experiments below. Sex was distinguished by colour of the matured portion of gametophytes (male: yellowish-brown; female: yellowish-green)^20^, since these colours reflect size of gametes formed. We confirmed the mating type of gametes by a crossing test.

### Microscopic observation

For fluorescence observation, gametic nuclei in each gametangium were stained with a DNA-localizing fluorochrome DAPI (4′-6-diamidino-2-phenylindole) (0.5 μg·ml^−1^) for 10 min after gametangia were fixed with 1% glutaraldehyde just before releasing of gametes^19^. Gametic nuclei were observed after individual gametangia were pressed between a cover slip and a microscopic slide. Released gametes were also fixed with 1% glutaraldehyde. Fixed gametangia and gametes were observed on a cover glass coated with poly-L-lysine (0.01 w/v%). We used an epifluorescence microscope (Axio Imager A1, Zeiss) for microscopic observation.

### Biovolume estimation for gametangium cells and gametes

**Figure 6:**
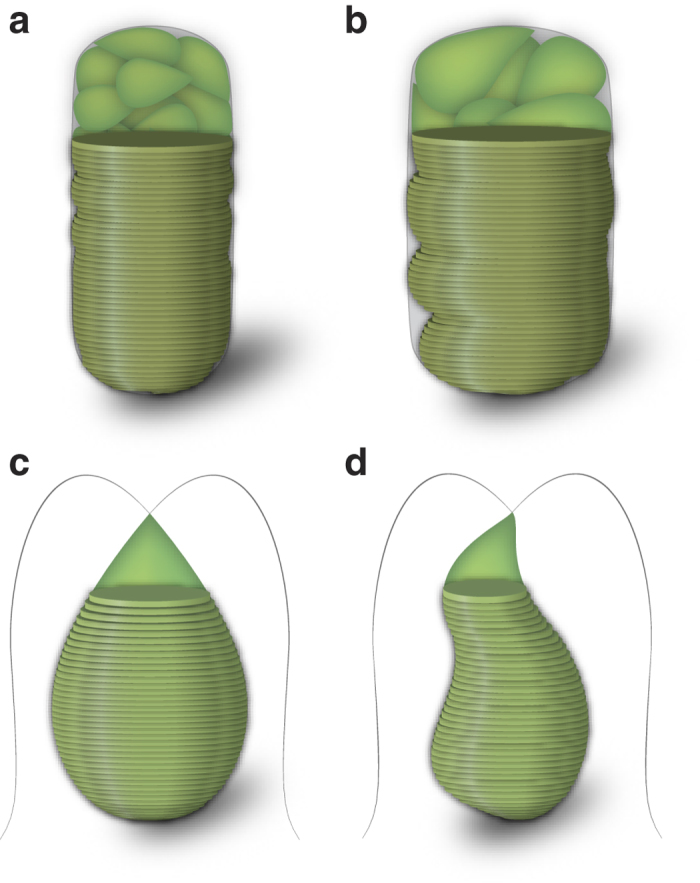
Schematics of calculation of cell volumes. (**a**) A gametangium cell that is symmetrical relative to the long axis. (**b**) A gametangium cell that is asymmetrical relative to the long axis. (**c**) A gamete that is symmetrical relative to the long axis. (**d**) A gamete that is asymmetrical relative to the long axis.

We more accurately estimated the volume of each gametangium cell and gamete than previous studies (see ref. 29 for review). In the conventional biovolume estimates for algae from microscopically measured linear dimensions, the calculation was based on geometric approximation: algal shapes were assumed as spheres, cylinders and ellipsoids etc. or combinations of these shapes. We eliminated the error due to discrepancies between these geometric shapes and real shapes without damage to live cells. In this study, gametangium cells and gametes of both mating types were usually cylindrical (Fig. 1) and pear-shaped (Fig. 4), respectively. We calculated the volume of each gametangium cell and gamete as follows (see also Fig. 6):

1. We obtained a vertical cross section of each cell based on its micrograph using a pen tablet (Wacom).
2. We calculated the volumes of each column with 1 pixel height.
3. We summed up the volumes of each column above to obtain the whole volume of the cell.

The pixel scale was calibrated with an object micrometer. We wrote a computer program using OpenCV (Itseez) and compiled the program using Visual C + + Express (Microsoft). See also Supplementary Note with Supplementary Figs 1 and 2 for the accuracy of this method.

### Comparison of predicted and measured gamete sizes

First, we measured the size of gametangia and examined the number of gametes formed in each gametangium. Each gametangium was separated by soft pipetting after the mature part of the gametophytes were fragmented. Gametogenesis had been completed in these gametangia because gametes were released several minutes later, if not fixed for measurement. Therefore, the number of gametic nuclei counted in an individual gametangium was equal to the number of gametes formed in that gametangium. The volume of each gametangium was calculated using the computer program that we developed above (Fig. 6a,b). The number of gametes formed in each gametangium was counted by staining gametic nuclei^19^. Based on these data, we predicted the gamete size of each mating type assuming various ratios of cell divisions (i.e. 5:5 ratio [equal], 4:6, 3:7, 2:8, and 1:9 [unequal]).

Second, we directly measured gamete size in each mating type. Live mature gametangia were desiccated and rehydrated under light to induce releasing of gametes^25^. The volume of each released gamete was similarly calculated using the computer program (Fig. 6c,d). Finally, we compared the distribution of directly measured gamete size with predicted distributions, assuming various ratios of cell divisions.

### General statistical analyses

We compared the two distributions represented by the sample data considering both of the positions and shapes. To avoid the effect of sample size, we compared the mean of measured gamete size with predicted gamete size assuming equal size cell divisions in each mating type, randomly choosing 100 gametes. Similarly, we took the effect of sample size into account to compare the variances. We used the Moses test for equal variability developed for contrasting the variances of two independent samples^21^. This is a nonparametric test of dispersion, which is recommended when there is reason to believe that the normality assumption required for parametric tests is violated as in this study. Note that if there is no statistically significant difference between two variables, this does not mean that there is no difference between them^30^.

## Additional Information

**How to cite this article**: Togashi, T. *et al*. Evidence for equal size cell divisions during gametogenesis in a marine green alga *Monostroma angicava*. *Sci. Rep*. **5**, 13672; doi: 10.1038/srep13672 (2015).

## Supplementary Material

Supplementary Information
