# Supplementary material for: Evidence for equal size cell divisions during gametogenesis in a marine green alga Monostroma angicava
Source: Sci Rep. 2015 Sep 3;5:13672. doi: 10.1038/srep13672 (PMC4558599; doi:10.1038/srep13672)
Supplement: Supplementary Information [file srep13672-s1.pdf]

Supplementary Information

Evidence for equal size cell divisions during gametogenesis in a marine green alga *Monostroma angicava*

Tatsuya Togashi, Yusuke Horinouchi, Hironobu Sasaki and Jin Yoshimura

Supplementary Notes

We checked whether our method to estimate the volume of each gametangium cell and gamete, using digital imaging and software (see Methods for more details), was appropriate. We compared our method with another conventional one for algae, where calculations of biovolume were made based on geometric approximation[29]. First, we mathematically produced 3 solid figures, which were similar to the above cells in shape, by rotating the cross sections (Supplementary Fig. S1a-c) around the Z axis (Supplementary Fig. S1d-f). In each figure, the population of the cells ranged from 5 μm to 10 μm in length at intervals of 0.1 μm. We mathematically calculated the real volume of each cell. Second, we estimated the volume of each cell using the computer program. Third, we geometrically approximated the volume of each cell as that of a cone and half sphere. Fourth, we similarly approximated the volume of each cell as that of an ellipsoid. Finally, we compared the real volume with the volume estimated using the computer program and the volumes geometrically approximated in each example (Supplementary Fig. S2). The volumes estimated using the computer program were not significantly different from the real volumes in all examples. In contrast, the geometrically approximated volumes were significantly different from the real volumes in some cases. Our method for biovolume estimation appears to be generally more accurate than the previous method in this study.

Supplementary Figure S1

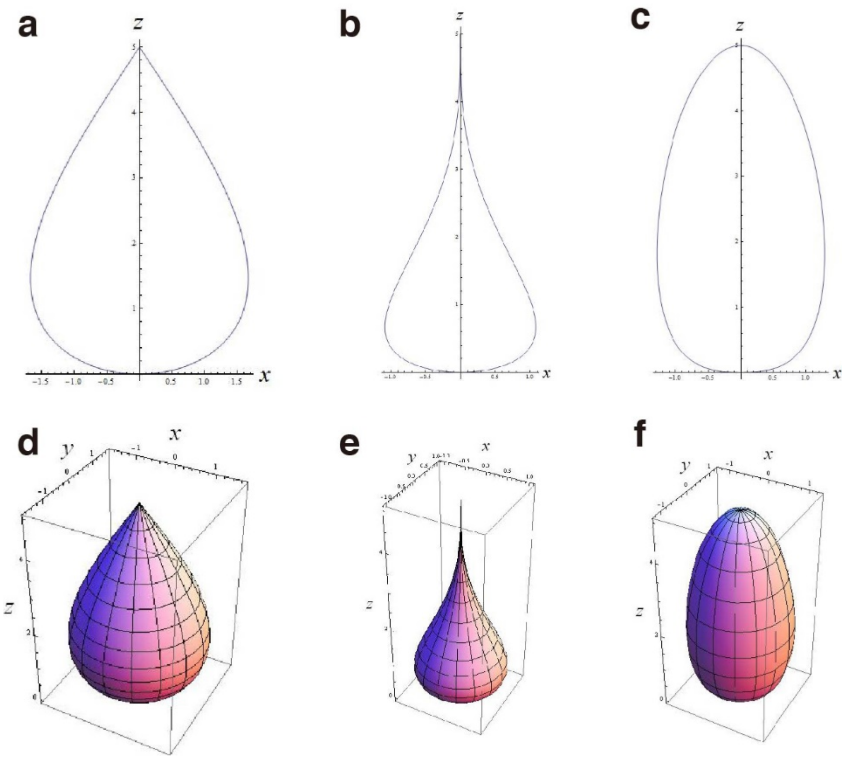

Supplementary Figure S1 | Mathematically produced artificial test cells. (a) A cross section produced by an equation, $\left(x(t),\ z(t)\right) = \left(\frac{5}{3}\sin(2t),\ 5(1-\cos(t))\right), \quad -\frac{\pi}{2} \le t \le \frac{\pi}{2}$. (b) A cross section produced by an equation, $\left(x(t),\ z(t)\right) = \left(\frac{5}{3}\sin(2t)\cos^2(t),\ 5(1-\cos(t))\right), \quad -\frac{\pi}{2} \le t \le \frac{\pi}{2}$. (c) A cross section produced by an equation, $\left(x(t),\ z(t)\right) = \left(\frac{5}{3}\sin(2t)\cos(t),\ 5\cos^5(t)\right), \quad -\frac{\pi}{2} \le t \le \frac{\pi}{2}$. (d) A solid figure produced by rotating the cross section (a) around the Z axis. (e) A solid figure produced by rotating the cross section (b) around the Z axis. (f) A solid figure produced by rotating the cross section (c) around the Z axis.

Supplementary Figure S2

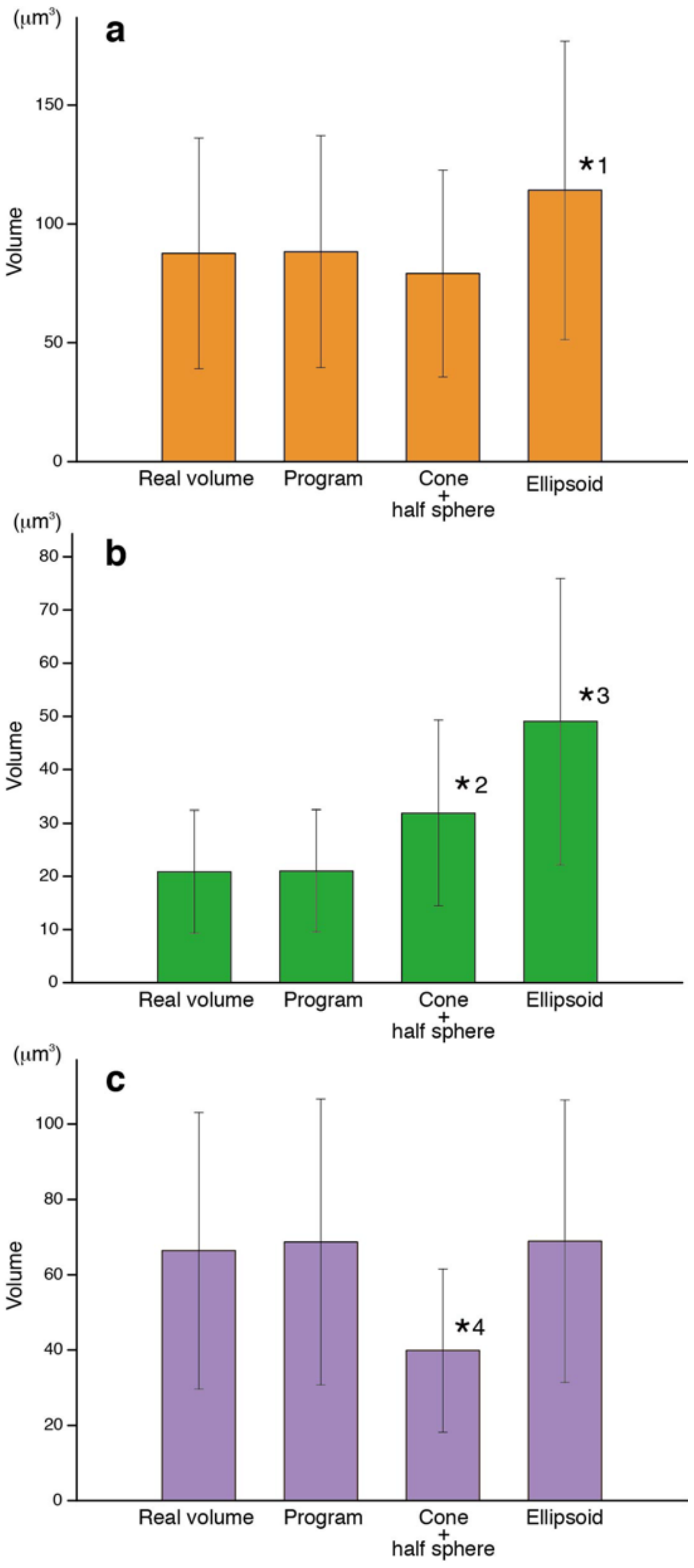

Supplementary Figure S2 | Comparison of the volumes of the test cells. The real volume calculated mathematically was compared with the volumes estimated using our computer program, the volume geometrically approximated as a cone and half sphere and the volume geometrically approximated as an ellipsoid. mean±SD. n=51 in each comparison. (a) The cell population was produced by Supplementary Fig. S1a. (b) The cell population was produced by Supplementary Fig. S1b. (c) The cell population was produced by Supplementary Fig. S1c. All the volume distributions of gametes departed significantly from normality at 5% level (Chi-square goodness-of-fit test) [(a) real volume: p=0.004; program: p=0.003; cone+half sphere: p=0.009; ellipsoid: p=0.009. (b) real volume: p=0.004; program: p=0.011; cone+half sphere: p=0.008; ellipsoid: p=0.008. (c) real volume: p=0.004; program: p=0.004; cone+half sphere: p=0.002; ellipsoid: p=0.004.]. *1~4: significantly different from the real volume at 5% level (*1: p=0.036; *2: p=0.0012; *3: $p=1.0\times10^{-8}$; *4: p=0.00016, Mann-Whitney U test).
